# Optimization of hydrogen production in *Enterobacter aerogenes* by Complex I peripheral fragments destruction and *maeA* overexpression

**DOI:** 10.1186/s12934-023-02155-6

**Published:** 2023-07-26

**Authors:** Ke Jiang, Ruoxuan Bai, Ting Gao, Ping Lu, Jingya Zhang, Shuting Zhang, Fangxu Xu, Shenghou Wang, Hongxin Zhao

**Affiliations:** 1 Zhejiang Province Key Laboratory of Plant Secondary Metabolism and Regulation, College of Life Sciences and Medicine, Zhejiang Sci-Tech University, Hangzhou, 310018 China; 2 Shenyang Functional Cordyceps militaris Industrial Technology Research Institute, Shenyang, 110034 China; 3 Liaoning Province Key Laboratory of Cordyceps Militaris with Functional Value, Experimental Teaching Center, Shenyang Normal University, Shenyang, 110034 China

**Keywords:** Hydrogen production, *Enterobacter aerogenes*, NADH dehydrogenase, NADH/NAD^+^ pool, Dark fermentation

## Abstract

As a concentrated energy source with high added value, hydrogen has great development prospects, with special emphasis on sustainable microbial production as a replacement for traditional fossil fuels. In this study, λ-Red recombination was used to alter the activity of Complex I by single and combined knockout of *nuoE*, *nuoF* and *nuoG*. In addition, the conversion of malic to pyruvic acid was promoted by overexpressing the *maeA* gene, which could increase the content of NADH and formic acid in the bacterial cells. Compared to the original strain, hydrogen production was 65% higher in the optimized strain IAM1183-EFG/M, in which the flux of the formic acid pathway was increased by 257%, the flux of the NADH pathway was increased by 13%, and the content of metabolites also changed significantly. In further bioreactor, the total hydrogen production of the scale-up IAM1183-EFG/M after 44 h of fermentation was 4.76 L, which increased by 18% compared with the starting strain. This study provides a new direction for future exploration of microbial hydrogen production by combinatorial modification of multiple genes.

**Graphical Abstract:**

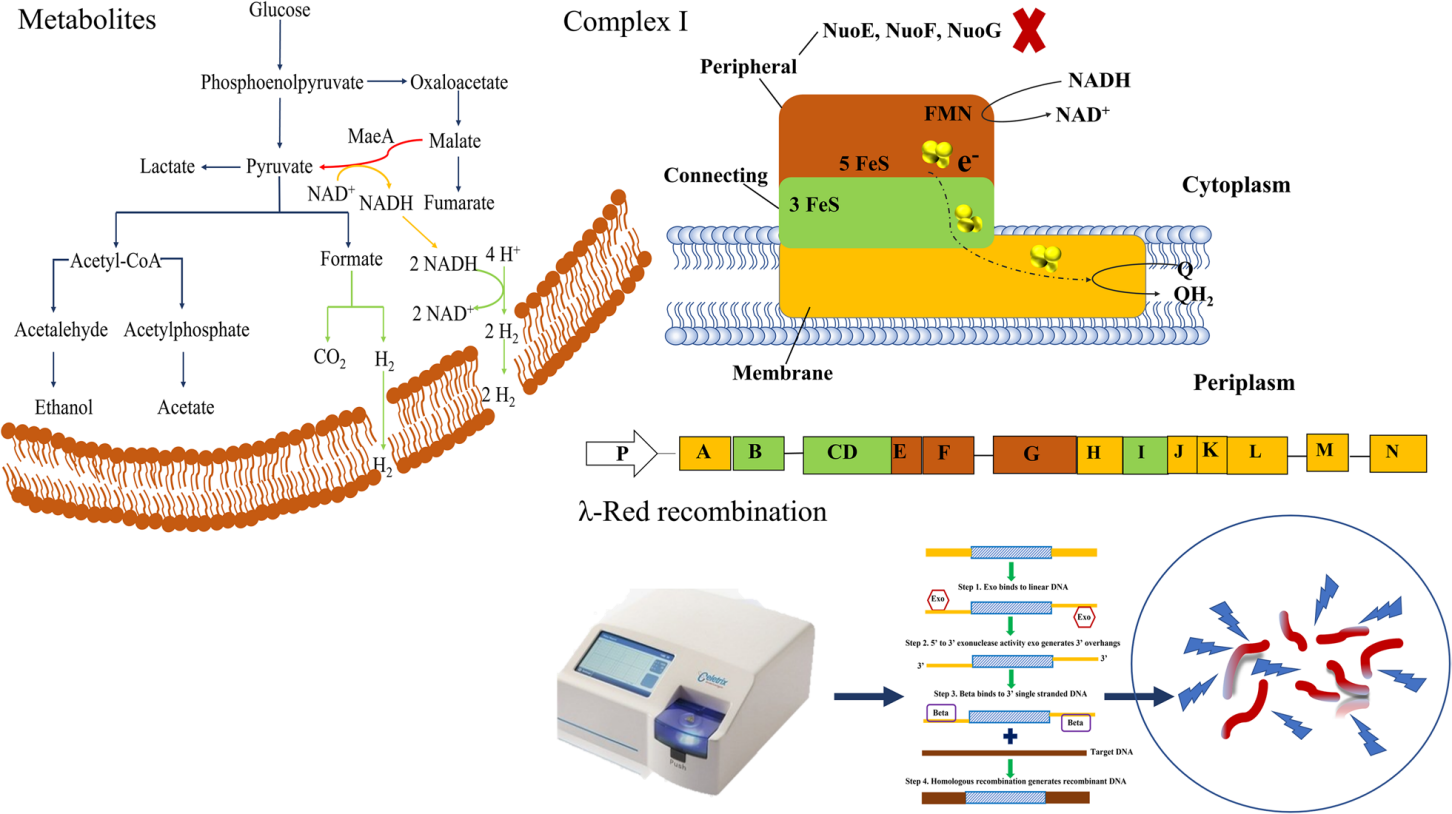

**Supplementary Information:**

The online version contains supplementary material available at 10.1186/s12934-023-02155-6.

## Introduction

The depletion of fossil fuels and the environmental problems caused by their use necessitate the urgent development of new and alternative environmentally friendly energy sources [1]. Standing out among new energy sources, hydrogen is a completely clean fuel because its combustion only produces water while offering high energy density [2, 3]. However, despite the enormous development potential of hydrogen energy, the production and utilization of hydrogen still faces many challenges. At present, hydrogen is mainly produced via the electrolysis of water and the high-temperature cracking of fossil fuels [4], both of which are not only energy-intensive and have low conversion rates, but also pollute the environment. In terms of economy and environmental protection, they are not suitable for long-term industrial hydrogen production [5]. Compared with traditional thermochemical hydrogen production technology, biotechnological hydrogen fermentation is a more promising hydrogen production process, which does not require harsh conditions and requires only inexpensive equipment [6, 7].

As a facultative anaerobe, *Enterobacter aerogenes* has strong adaptability to the environment and can produce hydrogen at a high conversion rate under strictly anaerobic conditions [8]. In addition, *E. aerogenes* also has the advantages of fast growth, wide substrate utilization range, easy cultivation and mature genetic modification methods. As a consequence, it is considered an ideal strain for large-scale biological hydrogen production. However, due to the incomplete metabolic process, the actual hydrogen production is far lower than the theoretical 4 mol H_2_/mol glucose. Therefore, understanding and optimizing the metabolic network of *E. aerogenes* is crucial for hydrogen production [9].

During dark fermentation for hydrogen production, *E. aerogenes* produces hydrogen through the formate pathway and the NADH pathway [7, 10]. In the formate pathway, pyruvate is converted by pyruvate formate lyase (*pfl*) to produce acetyl coenzyme A and formic acid. As the accumulation of formic acid leads to a decrease of extracellular pH, formic acid is oxidized to CO_2_ by formate dehydrogenase (FHL), and the generated electrons are transferred to hydrogenase, where they react with protons to form hydrogen. In theory, the maximum hydrogen conversion rate of the formic acid cleavage pathway is 2 mol H_2_/mol glucose [11]. In the NADH pathway, the hydrogen production process is mainly driven by the release of excess electrons or energy formed during anaerobic respiration. In this case, NADH mainly acts as an electron donor [12]. Therefore, if the distribution of NADH in *E. aerogenes* can be regulated, the hydrogen production level can be improved [13].

Redox reactions are the most common type of chemical reaction occurring in organisms, and are also common and extremely important reaction in biotransformations [14]. As intracellular cofactors, NADH and NAD^+^ not only affect the regulation of intracellular enzymes, but can also influence the rate of substrate conversion by organisms [15]. Cofactor engineering can effectively change the intracellular NADH or NAD^+^ status through the elimination of endogenous cofactor metabolic pathways, cofactor regeneration and competitive utilization, thus affecting the metabolic network, signal transduction and material transport.

As the key enzyme of hydrogen production in the NADH pathway, NADH dehydrogenase couples the oxidation of NADH with the generation of the proton motive force. A DNA probe prepared by PCR was used to identify its locus, and revealed that it was composed of 14 subunits, called *nuoA-nuoN* [16, 17]. If the L-shaped model is used to approximate Complex I, it can be found that it consists mainly of peripheral segments, connecting segments, and membrane-integrated segments. The peripheral segments encoded by the nuclear homologues *nuoE*, *nuoF* and *nuoG* can oxidize NADH to NAD^+^ [18–20]. Thus, if a single or multigene knockout is performed on the peripheral segment, it is likely to destabilize the complete structure, thus changing the distribution of NADH.

Succinic acid is one of the main products of anaerobic metabolism in *E. aerogenes*. Its production will not only convert a large amount of NADH into NAD^+^ [8], but also cause the culture medium to gradually acidify, which ultimately affects the normal growth of the strain. In the dark fermentation process of *E. aerogenes*, succinic acid is mainly produced from fumaric acid, which in turn generated from malic acid. As the key gene of NAD^+^ dependent malic acid enzyme, the transcriptional level of *maeA* can effectively modulate the conversion of malate into pyruvate [21], thus reducing the production of succinic acid. In addition, the pyruvate generated by *maeA* can also be used for formic acid production, increasing the hydrogen production rate of the formate pathway. At the same time, a large amount of NADH will be generated during the conversion of malic acid into pyruvate [22], which also lays a solid foundation for improving the hydrogen yield of the NADH pathway.

In this study, *E. aerogenes* IAM1183 was used as the starting strain. The *nuoG*, *nuoEF* and *nuoEFG* genes were knocked out by λ-Red recombination to create single- and multigene mutants, so as to compare and explore the effect of the deletion of *nuoG* on the overall hydrogen production of the strain. The effect of *nuoG* deletion on the hydrogen production through the NADH pathway was assessed by knocking out the *pfl* gene encoding pyruvate formate lyase, a key enzyme of the formate pathway. In addition, to further regulate anaerobic metabolism and enhance hydrogen production, In addition, the *maeA* gene encoding NAD^+^-dependent malic enzyme from *Escherichia coli* was cloned and heterologously expressed in *E. aerogenes* IAM1183 and its mutants to regulate intracellular NADH/NAD^+^ and promote hydrogen production through both the formate and NADH pathways. The strain with the highest hydrogen production was then tested in bioreactor scale-up to assess its potential for industrial production. These results further validate the effect of the *nuoG* and *maeA* genes on hydrogen production, and how the regulation of the NADH/NAD^+^ pool affects the metabolism of hydrogen-producing bacteria.

## Materials and methods

### Strains and plasmids

The strains and plasmids used in this study are shown in Table 1. Most of the strains were constructed in this study, except for the staring strain *E. aerogenes* IAM1183, *E. coli* DH5α and *E. coli* BL21 (DE3) [23].

### Culture conditions and source of reagents

*Enterobacter aerogenes* IAM1183 and its mutants were grown in Luria Bertani medium (per liter: 0.5% yeast extract, 1% trypsin and 1% NaCl; 1.5−2% agar powder was used for the preparation of solid plates) at 37 ℃ or 30 ℃ [24, 25]. The medium used for anaerobic fermentation contained (per liter): 1.5% glucose, 0.526% trypsin, 1.126% K_2_HPO_4_, 0.632% KH_2_PO_4_, 0.211% (NH_4_)_2_SO_4_ and 0.021% MgSO_4_ · 7H_2_O [26]. Unless otherwise specified, the LB medium contained 50 mg/L chloramphenicol or 50 mg/L kanamycin as appropriate.

### Construction of gene knockout strains

All primers used in this study were designed based on sequences from NCBI and synthesized by Sangon Bioengineering Co., LTD (Shanghai, China). The specific information is listed in Table 2. The plasmid DNA was purified using the DP103-03 plasmid extraction kit (Tiangen Biotech Co., Ltd.) to obtain the pKD46, pCP20 and FRT plasmids. The whole genome DNA of *E. aerogenes* was extracted using the Bacterial DNA Kit (Shanghai Qiming Biotech Co., Ltd.). The Gibson Assembly premix kit, all restriction endonucleases, DNA polymerase and DNA modification enzymes were purchased from Nanjing Weize Biotechnology Co., Ltd. (Nanjing, China).

**Table 1:** Primers were designed for recombinant plasmid construction in this study

Strain or plasmid	Genotype and relevant characteristics	Source or literature
IAM1183	*E. aerogenes* IAM1183	This study
IAM1183-G	*ΔnuoG*	This study
IAM1183-EF	*ΔnuoEF*	This study
IAM1183-EFG	*ΔnuoEFG*	This study
IAM1183-P	*Δpfl*	This study
IAM1183-GP	*ΔnuoG/Δpfl*	This study
IAM1183-EFP	*ΔnuoEF/Δpfl*	This study
IAM1183-EFGP	*ΔnuoEFG/Δpfl*	This study
IAM1183/M	carrying pET-28a- *maeA*	This study
IAM1183-G/M	*ΔnuoG* ,carrying pET-28a- *maeA*	This study
IAM1183-EF/M	*ΔnuoEF* ,carrying pET-28a- *maeA*	This study
IAM1183-EFG/M	*ΔnuoEFG* ,carrying pET-28a- *maeA*	This study
IAM1183-P/M	*Δpfl*, carrying pET-28a- *maeA*	This study
IAM1183-GP/M	*ΔnuoG/Δpfl*, carrying pET-28a-*maeA*	This study
IAM1183-EFP/M	*ΔnuoEF/Δpfl*, carrying pET-28a- *maeA*	This study
IAM1183-EFGP/M	*ΔnuoEFG/Δpfl*, carrying pET-28a- *maeA*	This study
*E. coli* DH5α	*F-, φ 80dlacZ ΔM15, Δ* (*lacZYA -argF*) *U169, deoR, recA1, endA1, hsdR17 (rK-, mK+), phoA, supE44, λ-, thi − 1, gyrA96, relA1*	TaRaKa
*E. coli* BL21(DE3)	*F* ^*−*^ *ompT hsdSB(rB* ^*−*^ *mB* ^*−*^ *) galdcm(DE3)*	TaRaKa
pET-28a	N-His, N-Thrombin, N-T7, C-His, Km	TaRaKa
pKD46	Cm	This lab
pCP20	Cm	TaRaKa
FRT	Km, FRT site	This study

As a gene editing technology that can precisely modify genes, λ-Red recombination completes a two-step homologous recombination process with the assistance of three plasmids [27]. According to its sequence of operation, the extracted plasmid pKD46 was electroporated into *E.aerogenes* IAM1183 at a voltage of 2500 V to express λ-Red recombinase. Subsequently, the FRT plasmid was used as a template, the corresponding forward, reverse primers and high-fidelity enzyme were used for PCR amplification to obtain the FRT homologous fragments that could be replaced with the genes to be knocked out, and then the same method was used to electroporate the constructs into cells containing pKD46 to induce homologous recombination. Under the action of the recombinant protein, the linear segment of the gene to be knocked out will cross with the FRT box, bringing the resistance gene into the host genome, which enables the selection of the gene deletion strain. Further sequencing authentication is required in order to verify the success of the transformation. In the positive strains, the pKD46 plasmid was eliminated by raising the culture temperature to 37 °C, after which the kanamycin resistance marker was eliminated by the pCP20 plasmid, which was electroporated into the verified positive strains. The elimination method of the pCP20 plasmid was the same as that of the pKD46 plasmid. The final mutant was submitted to Sangon Bioengineering Co., LTD for further sequencing certification.

### Construction of the *maeA* overexpression strain

The coding sequence of *maeA* was amplified by PCR using the genomic DNA of *E. aerogenes* as template with the maeA-F and maeA-R primers. Then, the linear gene fragments of the empty vector pET-28a and *maeA* were double digested with restriction endonucleases *Eco*RI and *Hin*dIII, and rejoined using T4DNA ligase to create the recombinant plasmid pET-28a-*maeA*. The newly created recombinant plasmid pET-28a-*maeA* was first electroporated into *E. coli* DH5α and BL21 [28], to determine the optimal expression conditions, after which the recombinant plasmid was electroporated into the gene deletion strain constructed above, and a final concentration of 0.1 mM isopropyl β-D-thiogalactoside (IPTG) was added to induce the overexpression of *maeA* [29].

### Determination of gene expression

To further investigate the effects of the deletion of *nuoG*, *nuoEF*, *nuoEFG*, *pfl* and the overexpression of *maeA* on dark fermentation in *E. aerogenes*, the *recA* gene was used as the internal reference. The key enzyme genes of ethanol dehydrogenase (*adh*), butanediol dehydrogenase (*bddh*), formate dehydrogenase (*fdh*), lactate dehydrogenase (*ldh*) and malate dehydrogenase gene (*mdh*) in the main by-product synthesis pathway were detected by qRT-PCR with corresponding primes [30]. RNA from all mutant strains was extracted using the PureLink™ RNA extraction kit [Thermo Fisher Technology (China) Co., Ltd.] and reversely transcribed into cDNA using the SuperScript IV Single Cell/Low-Input cDNA PreAmp kit [Thermo Fisher Technology (China) Co., Ltd.]. The extracted cDNA was used as the template for quantitative PCR using an ABI 7500 instrument (Shanghai Meixuan Biotechnology Co., LTD.), and the results were quantified using the 2^−△△CT^ method.

### Determination of NADH/NAD^+^

The intracellular NADH/NAD^+^ ratio was determined using a commercial detection kit (Abcam, Cambridge, UK). The standard curve was constructed by measuring the absorbance of NADH standard at 450 nm with an enzyme marker, and the test samples were prepared by using ultrasonication to lyse the cells, followed by vortexing. Finally, the NADH/NAD^+^ ratio of each sample was calculated according to the standard curve [31, 32].

### Small-scale anaerobic fermentation

Seed cultures were used to inoculate 100 mL serum bottles with a top cover, each bottle containing 20 mL of fermentation medium (5 g/L peptone, 10.7 g K_2_HPO_4_, 6 g KH_2_PO_4_, 2 g (NH_4_)_2_SO_4_, 0.2 g MgSO_4_·7H_2_O, 15 g glucose, glucose sterilized separately) [33], to an initial OD_600_ of calculated value. After inoculation, the culture medium was sparged with sterile nitrogen for 10 min to create an anaerobic environment. The prepared anaerobic cultures in serum bottles were incubated on a shaker at 37 ℃ and 200 rpm for 20 h, during which the pH and OD_600_ values of the cultures were measured every two hours, and at the end of 20 h, the total gas was collected with a syringe. After CO_2_ was absorbed by passing the gas through a 5 M sodium hydroxide solution, the remaining gas was defined as the total hydrogen produced by the anaerobic fermentation of the strain.

### Scale-up culture in a fermentation tank

The strain with the highest hydrogen production efficiency in small-scale anaerobic fermentationand the original strain were further tested in a 5 L fermentation tank (Lianyungang Hechang Bioengineering Equipment Co., Ltd). When the OD_600_ value of the seed culture reached 2, it was poured into 3 L of anaerobic fermentation medium at 37 ℃ and the anaerobic environment was maintained by sparging with sterile nitrogen gas for 1 h. The total incubation time was 44 h without feeding, during which the OD_600_ and pH changes of the culture were recorded in real time, and maintained at 37 ℃ and 200 rpm [34].

### Analytical methods

After the bacterial solution was properly diluted, the cell density (OD_600_) was measured using a UV-visible spectrophotometer (Spectrolab 752 S, Shanghai Precision Scientific Instrument Co., Ltd., China). The dry weight was obtained from the standard curve of DCW versus OD_600_ using the empirical formula: cell dry weight (g/L) = 0.132 × OD_600_ [35]. The residual content of glucose in the fermentation broth was determined colorimetrically using the 3,5-dinitrosalicylic acid (DNS) method, and the pH value was measured using a conventional pH meter (Cheng Du Sunge Instrument Co., Ltd.) [36]. Samples of the fermentation broth were collected every 4 h, centrifuged and filtered. After treatment, the content of formic acid, acetic acid, acetoin and other substances in the fermentation broth were determined by HPLC on an Aminex HPX-87 H column (Bio-Rad). The injection volume was 10 µL and the operating time was 25 min [37]. A gas chromatograph equipped with thermal conductivity detector (TCD) (GC112A, Shanghai Precision Scientific Instrument Co., Ltd., China) was used for gas analysis, with nitrogen as the carrier at a flow rate of 45 mL/min. The operating temperature of the column oven, injector and detector were set to 80 ° C, 90 ° C and 110 ° C respectively [38].

## Results and discussion

### Construction of mutant strains

The λ-Red recombination system was used to knock out the *nuoG*, *nuoEF*, *nuoEFG* and *pfl* genes. This strategy not only eliminates NADH dehydrogenase, but also makes it easier to observe the effect of the deletion of *nuo* genes on the NADH hydrogen production pathway by removing the formic acid hydrogen production pathway. All positive colonies were confirmed by sequencing after PCR analysis, and the results showed that the selected genes were successfully knocked out.

**Fig. 1:**
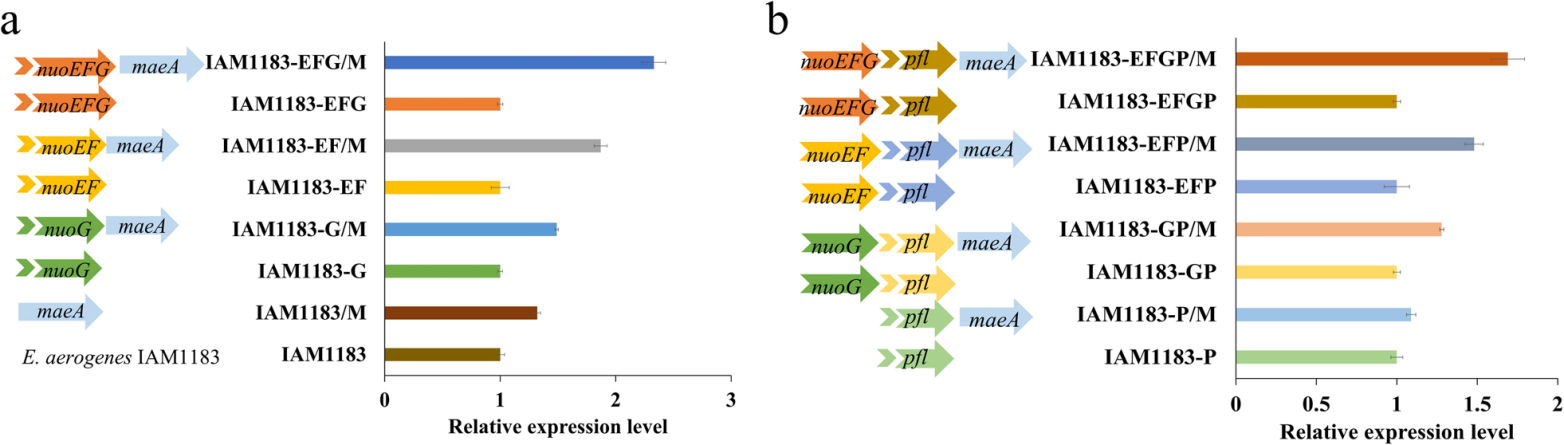
The relative expression levels of *maeA* genes. (**a** IAM1183, IAM1183-G, IAM1183-EF, IAM1183-EFG; **b** IAM1183-P, IAM1183-GP, IAM1183-EFP and IAM1183-EFGP)

In the overexpression experiment, to explore the optimal induction conditions required for the recombinant plasmid pET-28a-*maeA*, it was first transferred into *E. coli* BL21, and SDS-PAGE was used to analyze the protein expression. The results are shown in Additional file 1: Fig. S1. The strongest specific protein bands were observed when the cells were induced with 0.1 mM IPTG at 37 ℃ for 8 h, and these conditions were selected for further experiments. In the next step, the recombinant plasmids were electroporated into the previously obtained gene knockout strains, resulting in the eight different overexpression strains listed in Table 1. The increase in *maeA* expression levels of the strains was confirmed by qPCR, which confirmed a 2.3-fold increase in the IAM1183-EFGP/M strain. When the *pfl* gene was knocked out, the expression level of *maeA* decreased, but the overall trend remained the same (Fig. 1).

### Growth characteristics of mutant strains

**Fig. 2:**
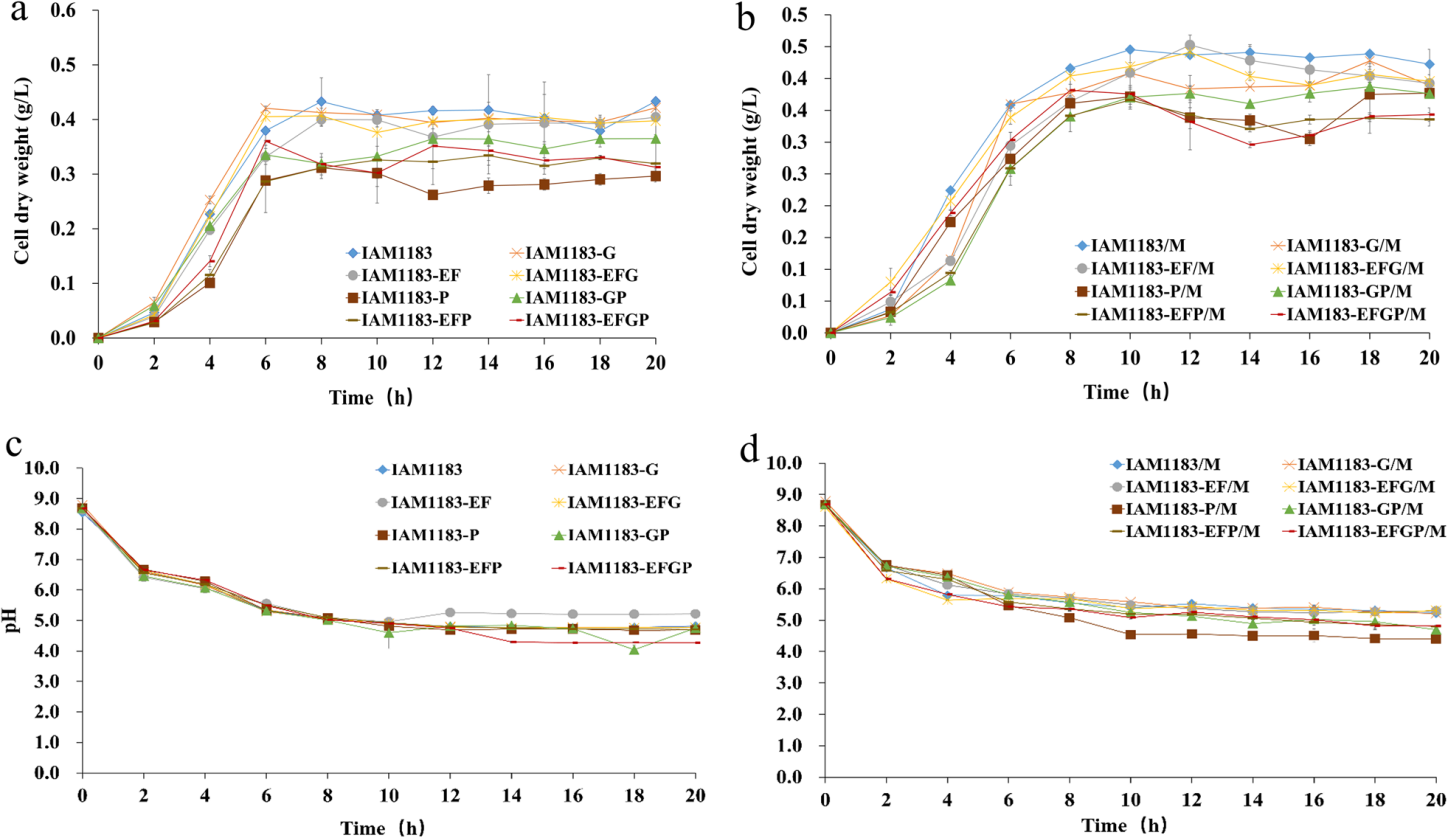
Growth status of 20 h anaerobic fermentation in serum bottles of the IAM1183 and its mutant strain {**a** Growth curve [in terms of cell dry weight (g/L)] of IAM1183, IAM1183-G, IAM1183-EF, IAM1183-EFG, IAM1183-P, IAM1183-GP, IAM1183-EFP and IAM1183-EFGP; **b** growth curve [in terms of cell dry weight (g/L)] of IAM1183/M, IAM1183-G/M, IAM1183-EF/M, IAM1183-EFG/M, IAM1183-P/M, IAM1183-GP/M, IAM1183-EFP/M and IAM1183-EFGP/M; **c** pH changes of IAM1183, IAM1183-G, IAM1183-EF, IAM1183-EFG, IAM1183-P, IAM1183-GP, IAM1183-EFP and IAM1183-EFGP; **d** pH changes of IAM1183/M, IAM1183-G/M, IAM1183-EF/M, IAM1183-EFG/M, IAM1183-P/M, IAM1183-GP/M, IAM1183-EFP/M and IAM1183-EFGP/M}

The wild-type *E. aerogenes* and its derived mutants were cultured anaerobically for 20 h in 100 mL serum bottles containing 20 mL of anaerobic fermentation medium to evaluate the effect of gene modifications on their fermentation characteristics. According to relevant research, gene mutation and protein expression tend to affect the normal physiological metabolism of bacteria, thus slowing down their growth. However,

during the first six h of cultivation, the cell dry weight values of *nuoG* and *nuoEFG* gene deletion strains were slightly higher than that of wild type strains, increasing by 10.8% and 6.7% respectively (Fig. 2a). By contrast, when the *nuoEF* and *pfl* genes were knocked out, the growth rate of the mutant strains in the logarithmic growth phase was slightly lower than that of the wild type strains, and the time to reach the stationary phase was also longer. After 20 h of fermentation, the final cell dry weight value of the original strain was the highest at 0.433 g/L, followed by the *nuoG*, *nuoEF* and *nuoEFG* deletion strains, while the mutant strain with *pfl* gene deletion exhibited the weakest growth (Fig. 2a). As *pfl* encodes formate lyase, its knockout increases the flow toward pyruvate to lactate, pyruvate decarboxylation and ethanol accumulation, resulting a slower growth rate (Table 3). This phenomenon also explains why the culture pH decreased after the *pfl* gene was knocked out (Fig. 2c).

**Table 2:** The production of metabolites and hydrogen after 20 h anaerobic fermentation in serum bottles of *Ea* and gene deficient strains

Primer for gene manipulation	Sequence (5′ to 3′)	Primer for gene qPCR verification	Sequence (5′ to 3′)
NuoG-F	CCCATGCTATCAACGGGATTCAGCCGAACCTGCTTAAGACGCGCTGGTAAAATTAACCCTCACTAAAGGGCG	T7	TAATACGACTCACTATAGG
NuoG-R	TTGAGGATGCTTAACAGAATGTCGATGAGATCCGGTGTTAACCAACTCATTAATACGACTCACTATAGGGCTC	T7-TER	GCTAGTTATTGCTCAGCGG
NuoEF-F	TCGTTTATCTGGGTAGTATCGATTTTGTTATGTCAGACGTG GACCGCTAAAATTAACCCTCACTAAAGGGCG	recA-f	ATTCTTTACGGCGAAGGTATCAA
NuoEF-R	TCCGCTCCGTTGACCTCGTATTCTTTGCCGTCTACATGAATCGTAGCCATTAATACGACTCACTATAGGGCTC	recA-r	CGGGTTATCTTTCAGCCACGA
NuoEFG-F	TCGTTTATCTGGGTAGTATCGATTTTGTTATGTCAGACGTG GACCGCTAAAATTAACCCTCACTAAAGGGCG	QmaeA-F	GGTTCTGGTAAGAGATAAAC
NuoEFG-R	TTGAGGATGCTTAACAGAATGTCGATGAGATCCGGTGTTAACCAACTCATTAATACGACTCACTATAGGGCTC	QmaeA-R	TCTGGTAAACAATCATCTTG
PFL-F	CAGCACTATGCTCAGAGCAAAGGTTTAACGGCTACTTTACGAGGATAAACTCAATTAACCCTCACTAAAGGGCG	Qadh-f	GAAGGCAGCCGTTGTTACCC
PFL-R	GAGCGGTGAATCCGTAGCCCGGCTAAGCGCAGCGCCAGCCGGGGGTTTTACTAATACGACTCACTATAGGGCTC	Qadh-r	CATACGCCACAGCACTCCATTT
G-F	GGGACTGACTTCCTGACCG	Qbddh-f	CACCGTGAACGCCTACTGCC
G-R	CAGCCGACACGGTTAGGT	Qbddh-r	ACCAGGCTCGCCACATCGTC
EF-F	GACTGCTATACCCGCGTGATG	Qfdh-f	CGTCCTGCAATACTTCGAGATG
EF-R	CGTCGTCGATAGAGATAAAGG	Qfdh-r	GAACTTCAGTTTCGACAGCGATT
EFG-F	GACTGCTATACCCGCGTGATG	Qldh-f	ACCAAAGCACGCAAGGTAATG
EFG-R	CAGCCGACACGGTTAGGT	Qldh-r	CCGTATCGAGGTCCACAAGGA
PFL-F	TAGGAGCTCTGAGCCGTTTATGAATCCTG	Qmdh-f	GCTGATGTGGTGCTGATTTCC
PFL-R	TGCTCTAGATACTGCTTGGCGTCATTG	Qmdh-r	GGCTTGCGGGCAGGTTTT
Check(cm)-F	AGTGCCAAGCTTGCATGCCT		
Check(cm)-R	AGTATACACTCCGCTAGCGC		
MaeA-F	CCGGAATTCAATGTTTCTTTTGACTACAT		
MaeA-R	CCCAAGCTTCTACAGCGATGAACGCTTATAGGAG		

Changes in the growth after overexpression of the *maeA* gene are shown in Fig. 2. The overall growth trend was the same as before overexpression, but the growth rate in the logarithmic growth phase was significantly lower than before. The mutants an additional 4 h to reach the stationary phase, indicating that overexpression of foreign proteins increased the growth burden of the strains (Fig. 2b). In addition, the pH value of the *maeA* overexpression strains decreased slightly (Fig. 2d), which may be due to the conversion of malic acid to pyruvate under the catalysis of the *maeA* gene. The increase in pyruvate content further promotes the generation of acidic substances such as formic acid and acetic acid, thereby reducing the pH of the fermentation broth (Table 4). These results suggested that overexpression of *maeA* caused changes in the metabolic flow of the strain, reducing the pH value of the fermentation broth and further affecting the growth of the strain.

**Table 3:** The production of metabolites and hydrogen after 20 h anaerobic fermentation in serum bottles of *Ea* and gene expressing strains

Strains	IAM1183	IAM1183-P	IAM1183-G	IAM1183-GP	IAM1183-EF	IAM1183-EFP	IAM1183-EFG	IAM1183-EFGP
Initial glucose (mM)	83.33	83.33	83.33	83.33	83.33	83.33	83.33	83.33
Residual sugar (mM)	38.99 ± 2.83	57.98 ± 1.05	37.65 ± 3.96	54.80 ± 0.97	33.73 ± 1.85	52.78 ± 1.32	36.53 ± 6.37	53.48 ± 2.37
Formic acid (mM)	14.01 ± 0.28	0	13.44 ± 0.27	0	15.58 ± 0.42	10.34 ± 0.27	14.9 ± 0.27	0
Lactic acid (mM)	1.02 ± 0.09	2.31 ± 0.01	3.90 ± 1.06	5.79 ± 0.10	3.26 ± 0.24	3.40 ± 0.32	2.87 ± 0.24	4.66 ± 0.01
Acetic acid (mM)	4.95 ± 0.53	1.57 ± 0.30	4.97 ± 0.09	2.62 ± 0.40	3.85 ± 0.43	4.47 ± 0.52	3.57 ± 0.92	1.53 ± 0.02
Citric acid (mM)	0.65 ± 0.10	0.57 ± 0.05	0.86 ± 0.04	0.53 ± 0.02	0.88 ± 0.05	0.15 ± 0.01	0.89 ± 0.03	0.79 ± 0.03
Acetoin (mM)	0.52 ± 0.16	0.49 ± 0.95	0.52 ± 0.16	0.72 ± 0.46	0.52 ± 0.16	0.86 ± 0.84	0.78 ± 0.10	0.59 ± 0.11
2,3-Butanediol (mM)	0	0	1.56 ± 0.31	0	1.13 ± 0.12	0	1.1 ± 0.22	0
Ethanol (mM)	2.72 ± 0.09	2.82 ± 0.05	2.66 ± 0.07	2.86 ± 0.88	2.36 ± 4.28	2.98 ± 0.92	1.58 ± 1.91	2.88 ± 0.57
H_2_ yield (mol/mol glucose)	1.34 ± 0.21	0.79 ± 0.19	1.61 ± 0.03	0.94 ± 0.04	1.66 ± 0.05	1.34 ± 0.21	1.54 ± 0.04	1.41 ± 0.03

**Table 4:** The production of metabolites and hydrogen after 20 h anaerobic fermentation in serum bottles of gene expressing strains

Strains	IAM1183/M	IAM1183-P/M	IAM1183-G/M	IAM1183-GP/M	IAM1183-EF/M	IAM1183-EFP/M	IAM1183-EFG/M	IAM1183-EFGP/M
Initial glucose (mM)	83.33	83.33	83.33	83.33	83.33	83.33	83.33	83.33
Residual sugar (mM)	32.50 ± 6.19	53.98 ± 2.13	35.16 ± 0.85	49.20 ± 0.87	29.65 ± 0.49	48.7 ± 1.73	36.42 ± 0.59	49.48 ± 1.02
Formic acid (mM)	15.70 ± 1.08	9.72 ± 2.49	16.49 ± 1.30	0	18.54 ± 1.30	5.62 ± 1.20	18.62 ± 0.83	0
Lactic acid (mM)	2.95 ± 0.28	2.87 ± 0.38	5.03 ± 0.12	3.79 ± 0.10	6.09 ± 0.03	2.88 ± 0.46	6.12 ± 0.05	3.66 ± 0.01
Acetic acid (mM)	5.65 ± 0.14	1.69 ± 0.15	6.49 ± 0.65	0.62 ± 0.40	5.37 ± 0.03	2.82 ± 0.83	4.29 ± 0.22	1.53 ± 0.02
Citric acid (mM)	0.11 ± 0.10	0.24 ± 1.71	0.16 ± 0.03	0.03 ± 0.02	0.59 ± 0.02	0.33 ± 0.05	0.28 ± 0.24	0
Acetoin (mM)	0.95 ± 0.52	0.97 ± 0.18	0.41 ± 0.16	1.22 ± 0.46	0.15 ± 0.26	4.68 ± 0.39	0.36 ± 0.15	0.59 ± 0.11
2,3-Butanediol (mM)	0.22 ± 0.13	0	1.96 ± 0.21	0	0.13 ± 0.48	0	2.51 ± 0.05	0
Ethanol (mM)	2.91 ± 0.56	2.61 ± 0.15	1.96 ± 0.21	2.66 ± 0.88	2.68 ± 0.80	4.68 ± 2.26	2.54 ± 0.05	1.68 ± 0.57
H_2_ yield (mol/mol glucose)	2.21 ± 0.09	1.19 ± 0.02	2.29 ± 0.07	1.67 ± 0.01	2.33 ± 0.09	1.64 ± 0.24	2.63 ± 0.03	1.59 ± 0.03

### Effect of genetic modification on the relative expression levels of key enzyme coding genes

**Fig. 3:**
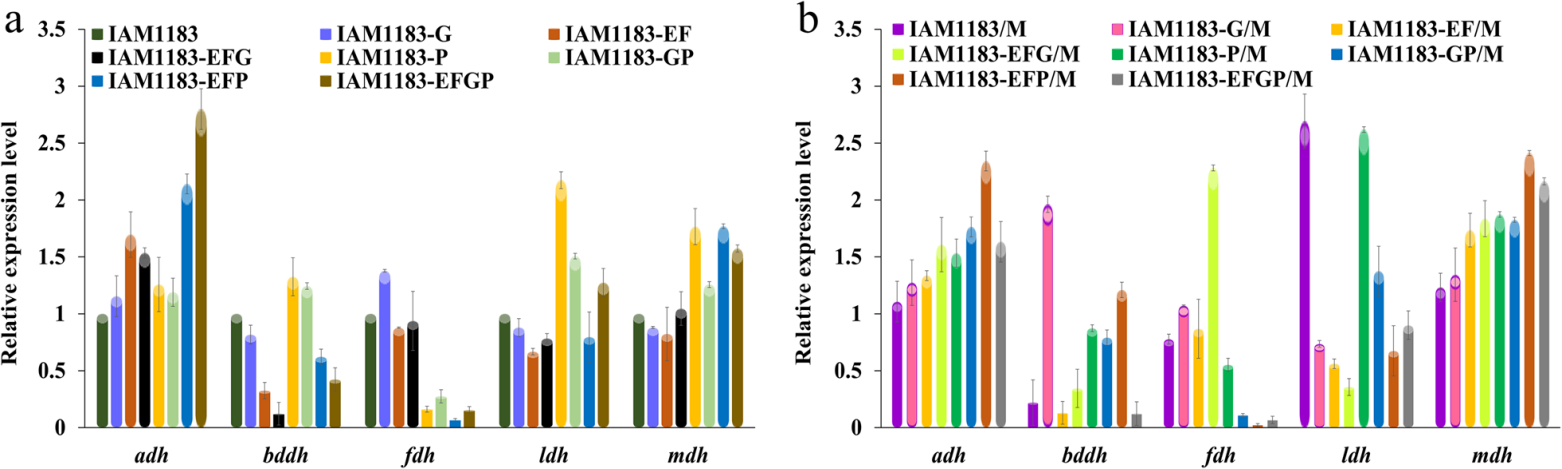
Comparison of the effects of gene modification on the expression levels of important genes in metabolic pathways (**a** IAM1183, IAM1183-G, IAM1183-EF, IAM1183-EFG, IAM1183-P, IAM1183-GP, IAM1183-EFP and IAM1183-EFGP; **b** IAM1183/M, IAM1183-G/M, IAM1183-EF/M, IAM1183-EFG/M, IAM1183-P/M, IAM1183-GP/M, IAM1183-EFP/M and IAM1183-EFGP/M)

In addition to producing hydrogen, *E. aerogenes* also produces various metabolic by-products when using glucose as carbon source for anaerobic metabolism, such as formic acid, lactic acid, succinic acid and ethanol. When the substrate is limited, the pathways can balance each other, thus affecting the flux of other pathways. Therefore, genetic modification can effectively reduce the generation of by-products and improve the yield of target products. The effect of gene modification on other metabolic fluxes can be effectively observed by performing quantitative PCR to determine the relative expression levels of the genes encoding other key enzymes of the anaerobic metabolic pathway (*adh*, *bddh*, *fdh*, *ldh* and *mdh*) in the original strain and mutants after gene knockout and overexpression. As shown in Fig. 3a, knockout of the *nuoEFG* gene, as the peripheral segment of Complex I, had a greater impact on other key genes. Except for the up-regulation of the relative expression level of *adh* and *mdh* genes, the expression of other genes was down regulated to varying degrees. In addition, as the key genes of the formate pathway, the expression levels of *adh*, *ldh* and *mdh* were significantly increased in the *pfl* gene deletion mutants, which also explained why the OD_600_ and pH values of these strains decreased (Fig. 2). However, after overexpression of the *maeA* gene, the expression level of the other key genes also changed significantly. Among them, the expression levels of *adh*, *fdh* and *mdh* in IAM1183-EFGP/M were significantly increased, with mdh expression being upregulated substantially in all overexpression mutants, while the expression levels of other genes fluctuated to varying degrees (Fig. 3b). These results reflect the highly complex anaerobic metabolism of *E. aerogenes*. As a result, the changes of metabolic flow caused by the modification of the strain at the molecular level can be unpredictable, the joint adjustment of the two metabolic branches does not necessarily change the metabolic flow in the desired direction.

### Effect of genetic mutation on the intracellular NADH/NAD^+^ ratio

**Fig. 4:**
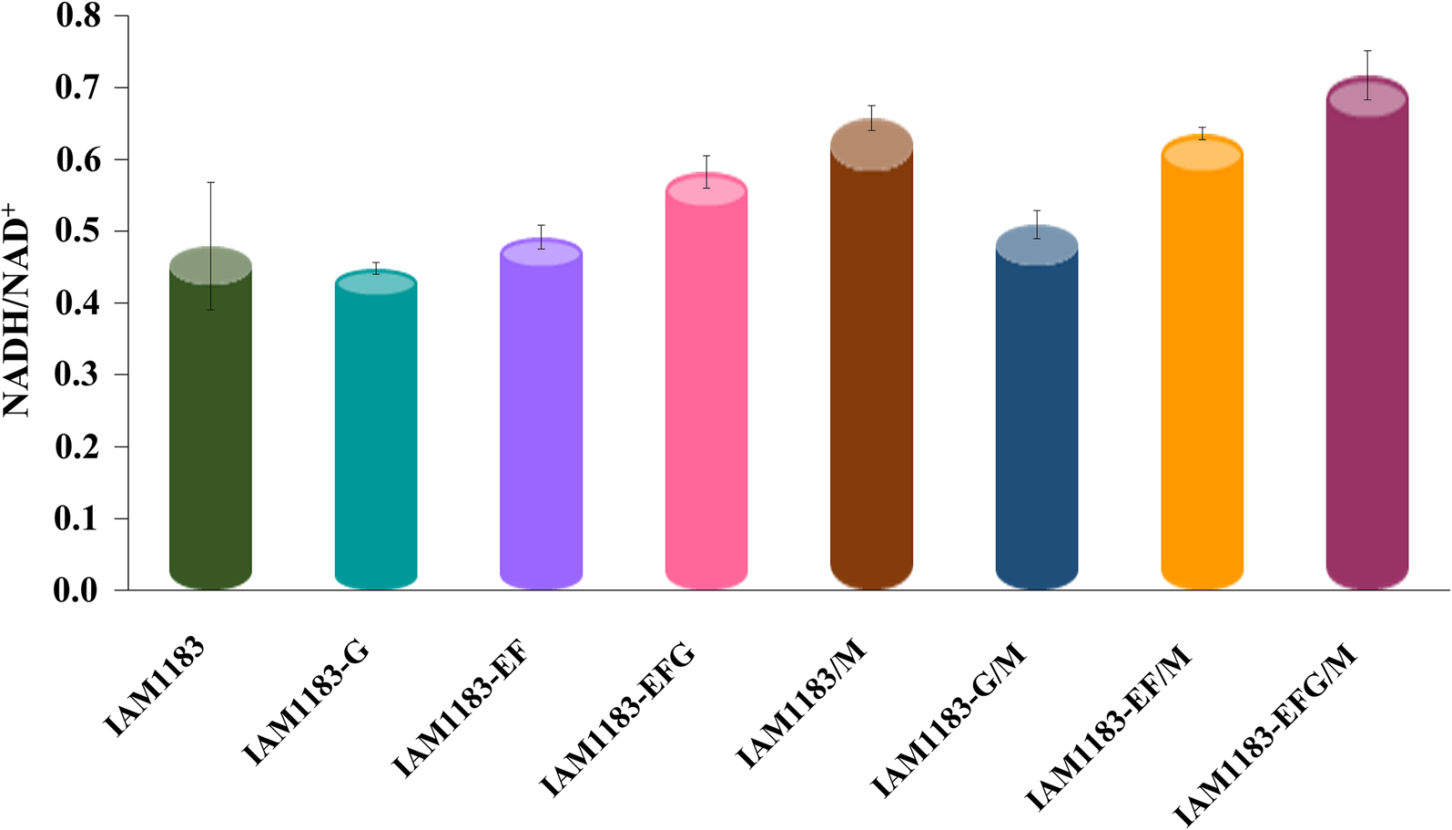
Intracellular NADH/NAD^+^ ratio of the IAM1183 and its mutant strains

Intracellular NADH can provide the required reducing power for metabolic pathways, while the NADH/NAD^+^ ratio reflects the intracellular redox state, which has important implications for further regulating NADH to enhance hydrogen production. As shown in Fig. 4, the knockout of *nuoG* and *nuoEF* did not cause a significant change in the NADH/NAD^+^ ratio, and even slightly decreased by 6.5% and 3.6%. However, the combined knockout of *nuoEFG* resulted in a significant 22% increase in the intracellular NADH/NAD^+^ ratio. This result may also be related to the formation of the peripheral fragments of *nuoEFG* controlling Complex I. In addition, the overexpression of the *maeA* gene also caused a significant fluctuation in the NADH/NAD^+^ ratio, resulting in an increase of 37%, 14%, 29%, and 23% compared to before expression. Among them, the most obvious change was observed in IAM1183/M, whose NADH/NAD^+^ ratio was 37%, higher than in the original strain, indicating that *maeA* has a positive effect on the intracellular NADH level. These results also indicated that combinatorial multigene editing may be a more powerful strategy for improving the performance of strains than single-gene mutation.

### Effect of genetic modification on hydrogen production

**Fig. 5:**
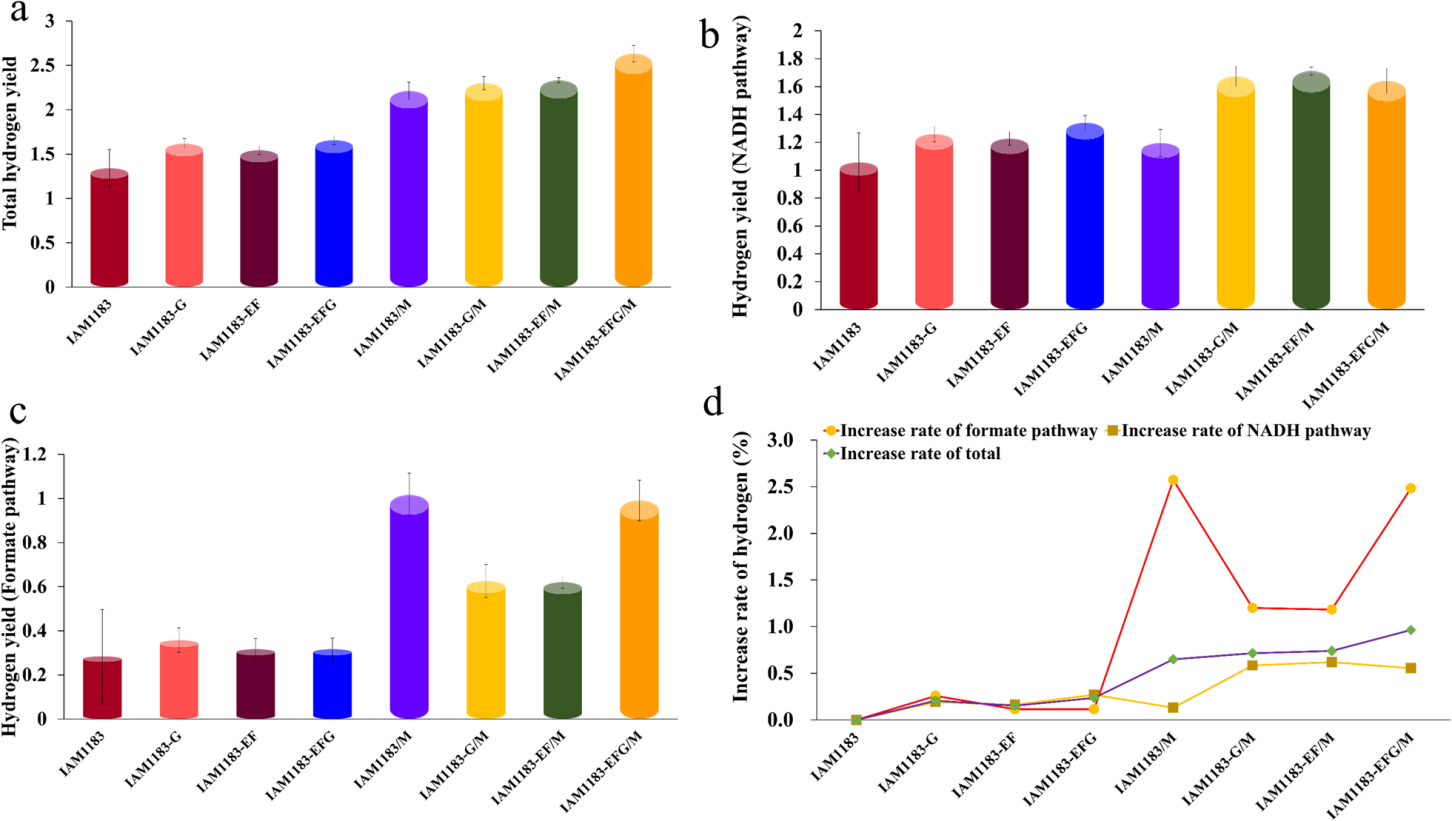
Comparison of hydrogen yield between the IAM1183 and its mutant strain in formate pathway and NADH pathway using glucose as substrate (**a** total hydrogen yield; **b **hydrogen yield (NADH pathway); **c** hydrogen yield (Formate pathway); **d **increase rate of hydrogen )

*E. aerogenes* can produce hydrogen either through the formate pathway or the NADH pathway. When related genes are modified, the metabolic flow in the cell will be affected or even redistributed, resulting in changes of hydrogen production in each pathway. In order to better determine the effect of gene modification on the NADH pathway, we analyzed the metabolite content following the knockout of the *pfl* gene to calculate hydrogen production in each pathway. As shown in Fig. 5, the total hydrogen production improved in all gene knockout strains, with IAM1183-G and IAM1183-EFG respectively increasing by 20% and 24% compared with the original strain (Fig. 5a). In addition, the hydrogen production of IAM1183-EFG, which was constructed by knocking out *nuoG* in IAM1183-EF, also slightly increased by 15% compared to the parental strain without the knockout. By contrast, when the *maeA* gene was overexpressed, the hydrogen production of the mutant significantly increased, and the largest change was related to increased hydrogen production in the formic acid pathway. In IAM1183/M, the total hydrogen production increased by 65% compared with the original strain (Fig. 5a and d), whereby the flux of the NADH pathway increased by 13% (Fig. 5b and d), while the flux of the formate pathway increased by a remarkable 251% (Fig. 5c and d), indicating that the overexpression of *maeA* is highly beneficial for hydrogen production in *E. aerogenes*. Furthermore, knocking out *nuo* genes in the overexpressed strain could further improve the hydrogen production rate, with IAM 1183-EFG/M showing the highest hydrogen production rate, reaching 2.63 mol H_2_/mol glucose, which was 74% higher than in the original strain.

### Scale-up culture of the best strains

**Fig. 6:**
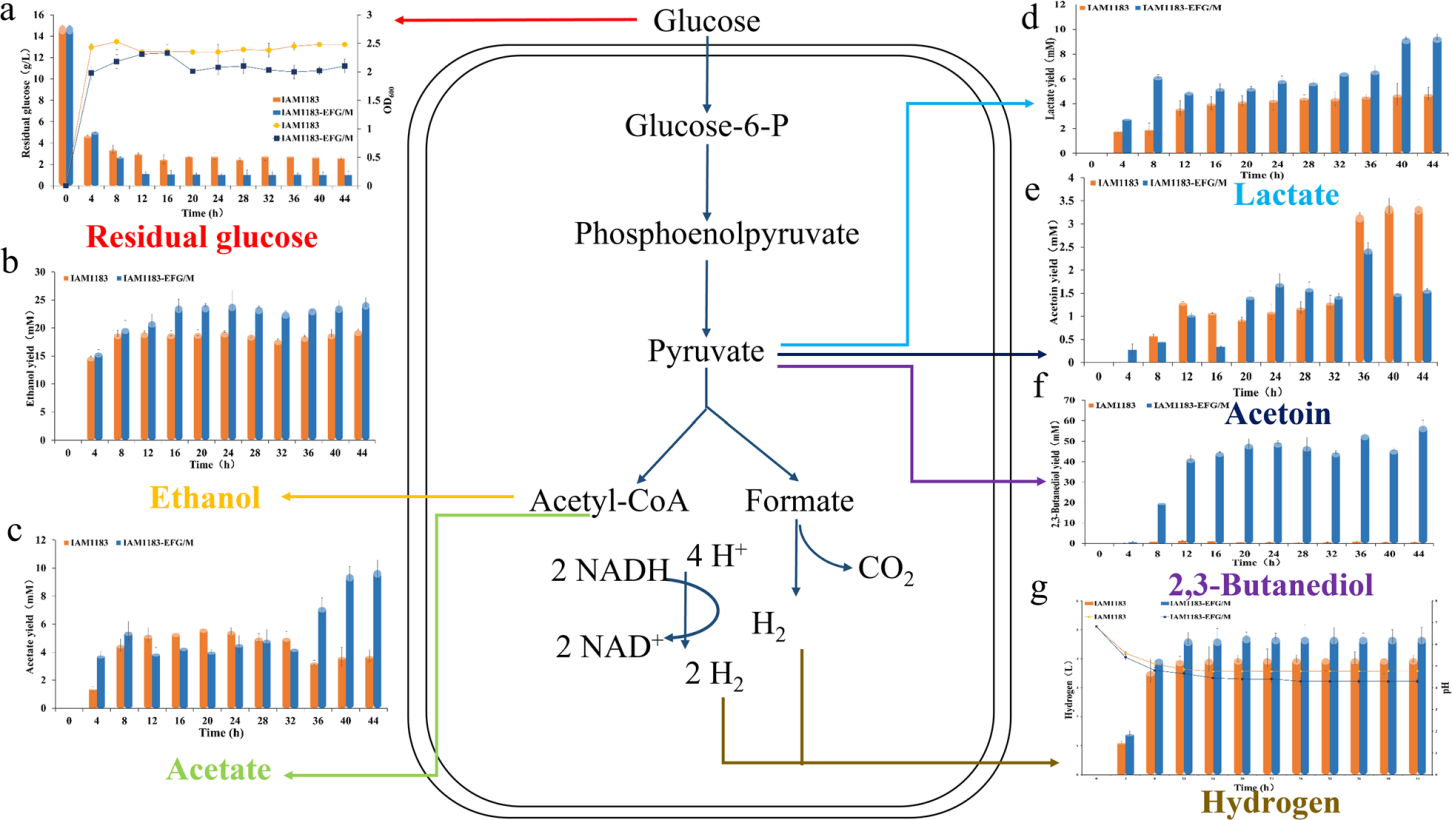
Metabolite changes of IAM1183 and IAM1183-EFG/M in a 5-L fermenter for 44 h of anaerobic fermentation {**a** Glucose consumption and growth curve [in terms of cell dry weight (g/L)]; **b** ethanol production; **c** acetate production; **d** lactate production; **e** acetoin production; **f** 2,3-Butanediol production; **g**. Hydrogen production and pH changes}

As shown in Fig. 5, IAM1183-EFG/M exhibited the best hydrogen production performance. Therefore, this strain was selected for scale-up cultivation in a 5-L fermentation tank and compared with the original strain IAM1183 to investigate its potential for industrial application. The growth and metabolite levels during anaerobic cultivation in the fermentation tank for 44 h are shown in the Fig. 6. In the first 4 h, the cell density of the two strains increased exponentially, and then gradually stabilized after 8 h. During this period, the growth of the mutant IAM1183-EFG/M was always slightly weaker than that of the parent strain, but its substrate utilization rate was significantly increased by 13%. In addition, the culture pH of IAM1183-EFG/M was significantly lower after 4 h (Fig. 6g), which was attributed to increased synthesis of acid by-products caused by the overexpression of the *maeA* gene, which affected the normal growth of the strain and the overall pH of the culture medium.

Further metabolite analysis revealed that the hydrogen production rate of the strain was the highest in the first 8 h of anaerobic growth. After 12 h, the hydrogen production gradually decreased, with the final total hydrogen production of the mutant strain reaching 4.76 L, which was 18% higher than that of the original strain (Fig. 6g). Based on the growth curve and glucose utilization rate, the first 8 h were optimal for hydrogen production by *E.aerogenes*. After 12 h, the growth of the strain will enter a relatively stable stage, at which time the hydrogen production will not fluctuate too much, which may be caused by the acidic environment of the fermentation medium and the competition of other metabolites. As for other by-products, a comparison with the parental strain showed that except for the low yield of acetoin, the others had significantly increased, including lactic acid, acetic acid and ethanol, which have increased by 97%, 165% and 25% respectively. This result is also in line with the characteristics of the weak growth trend of mutant (Fig. 6).

## Conclusions

In this study, we first studied the peripheral fragment genes of NADH dehydrogenase I and successfully increased the hydrogen production of *E.aerogenes* by the combined modification of multiple genes. Among them, *nuoE*, *nuoF* and *nuoG* are the key genes that encode the peripheral segment of NADH dehydrogenase. Their single and combined knockout can effectively change the activity of Complex I and improve hydrogen production in the NADH pathway. The most obvious change was observed in strain IAM1183-EFG, whose hydrogen production was 24% higher than that of the original strain. At the same time, there are few cases of overexpression of *maeA* in *E.aerogenes.* Through this study, it can be found that *maeA* not only increased the intracellular NADH level of the strain, but also increased the conversion rate of malate to pyruvate, as well as the production of formic and lactic acid, thus significantly improving the two hydrogen production pathways of *E. aerogenes*. This suggests that the strategic combination of genes is an effective method for increasing the hydrogen production of *E.aerogenes*. Future studies should focus on eliminating the synthesis of other by-products to further increase the hydrogen production of engineered strains.

## Supplementary Information

**Additional file 1: Figure S1.** *MaeA* expression detection on SDS-PAGE (1: IAM1183; 2: IAM1183-G; 3: IAM1183-EF; 4: IAM1183-EFG; 5: IAM1183-P；6: IAM1183-GP; 7: IAM1183-EFP；8: IAM1183-EFGP).

## Data Availability

The data of this study can be shared openly. All data can be obtained from the corresponding author or first author.
