# Supplementary material for: Optimization of hydrogen production in Enterobacter aerogenes by Complex I peripheral fragments destruction and maeA overexpression
Source: Microb Cell Fact. 2023 Jul 26;22:137. doi: 10.1186/s12934-023-02155-6 (PMC10373349; doi:10.1186/s12934-023-02155-6)
Supplement: Supplementary file 1 — Additional file 1: Figure S1. MaeA expression detection on SDS-PAGE (1: IAM1183; 2: IAM1183-G; 3: IAM1183-EF; 4: IAM1183-EFG; 5: IAM1183-P；6: IAM1183-GP; 7: IAM1183-EFP；8: IAM1183-EFGP). [file 12934_2023_2155_MOESM1_ESM.docx]

**Additional file 1**

**Figure S1**


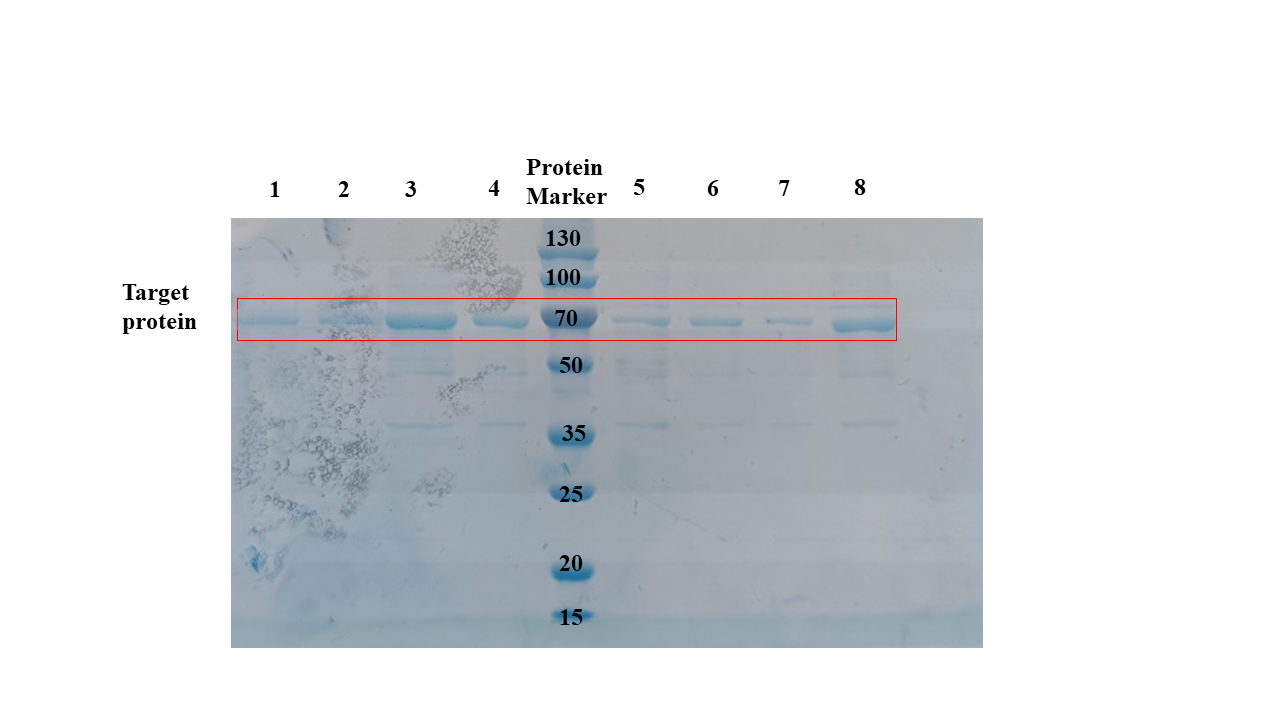


**Fig. S1 – *MaeA* expression detection on SDS-PAGE (1:** **IAM1183; 2: IAM1183-G; 3: IAM1183-EF; 4: IAM1183-EFG; 5: IAM1183-P；6: IAM1183-GP; 7: IAM1183-EFP；8: IAM1183-EFGP)**

The recombinant plasmid pET-28a-MaeA was transformed into IAM1183, IAM1183-G, IAM1183-EF, IAM1183-EFG, IAM1183-P, IAM1183-GP, IAM1183-EFP and IAM1183-EFGP strains. All mutants and wildtype IAM1183 were cultured in LB containing kanamycin (50 µg/mL) at 37 °C. Protein expression was induced with 0.1 mM (final concentration) isopropyl thiogalactoside (IPTG) for 8 h and checked by SDS-PAGE. According to the electrophoresis results, compared with the uninduced strain, the remaining mutant strains have obvious bands between 50-70 KDa. Since the size of the protein encoded by *MaeA* gene is about 67.4 kDa, so when the final result band was in this range, it could be judged that the overexpression was successful. The result was shown that the *MaeA* was successfully expressed in all mutant strains and wild type IAM1183 (Fig. S1).
